# 1-(1,3-Benzodioxol-5-yl)ethanone

**DOI:** 10.1107/S1600536810001352

**Published:** 2010-01-16

**Authors:** Jerry P. Jasinski, Ray J. Butcher, Q. N. M. Hakim Al-arique, H. S. Yathirajan, B. Narayana

**Affiliations:** a Department of Chemistry, Keene State College, 229 Main Street, Keene, NH 03435-2001, USA; b Department of Chemistry, Howard University, 525 College Street NW, Washington, DC 20059, USA; c Department of Studies in Chemistry, University of Mysore, Manasagangotri, Mysore 570 006, India; d Department of Studies in Chemistry, Mangalore University, Mangalagangotri 574 199, India

## Abstract

In the title compound, C_9_H_8_O_3_, the dihedral angle between the mean planes of the benzene and dioxole rings is 1.4 (8)°, with the dioxole group in a slightly distorted envelope configuration with the flap C atom displaced by 0.0645 Å from the plane through the other four atoms. In the crystal, weak inter­molecular C—H⋯O hydrogen-bond inter­actions link the mol­ecules into chains propagating in [011]. The crystal packing exhibits weak π–π inter­actions as evidenced by the relatively short distances [3.801 (9) Å] between the centroids of adjacent benzene rings.

## Related literature

For the pharmaceutical properties of compounds containing the 1,3-dioxolyl group, see: Gabrielsen *et al.* (1992 ▶); Krause & Goeber (1972 ▶); Ma *et al.* (1987*a* ▶,*b* ▶); Ohta & Kimoto (1976 ▶); For bond-length data, see: Allen *et al.* (1987 ▶). For related structures, see: Jasinski *et al.* (2008 ▶); Yathirajan *et al.* (2007 ▶). For puckering parameters, see: Cremer & Pople (1975 ▶). For MOPAC AM1 calculations, see: Schmidt & Polik (2007 ▶).
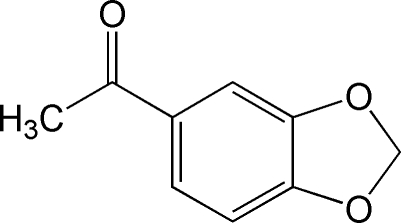


## Experimental

### 

#### Crystal data

C_9_H_8_O_3_
*M* *_r_* = 164.15
Monoclinic, 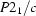

*a* = 9.4697 (3) Å
*b* = 10.8445 (3) Å
*c* = 7.5148 (3) Å
β = 105.973 (3)°
*V* = 741.93 (4) Å^3^
*Z* = 4
Mo *K*α radiation
μ = 0.11 mm^−1^
*T* = 200 K
0.58 × 0.45 × 0.26 mm

#### Data collection

Oxford Diffraction R Gemini diffractometer
Absorption correction: multi-scan (*CrysAlis RED*; Oxford Diffraction, 2007 ▶) *T*_min_ = 0.909, *T*_max_ = 0.972
12470 measured reflections
3061 independent reflections
2215 reflections with *I* > 2σ(*I*)
*R*_int_ = 0.024

#### Refinement

*R*[*F* ^2^ > 2σ(*F* ^2^)] = 0.045
*wR*(*F* ^2^) = 0.133
*S* = 1.03
3061 reflections
110 parameters
H-atom parameters constrained
Δρ_max_ = 0.39 e Å^−3^
Δρ_min_ = −0.28 e Å^−3^

### 

Data collection: *CrysAlis PRO* (Oxford Diffraction, 2007 ▶); cell refinement: *CrysAlis PRO*; data reduction: *CrysAlis PRO* program(s) used to solve structure: *SHELXS97* (Sheldrick, 2008 ▶); program(s) used to refine structure: *SHELXL97* (Sheldrick, 2008 ▶); molecular graphics: *SHELXTL* (Sheldrick, 2008 ▶); software used to prepare material for publication: *SHELXTL*.

## Supplementary Material

Crystal structure: contains datablocks global, I. DOI: 10.1107/S1600536810001352/im2174sup1.cif

Structure factors: contains datablocks I. DOI: 10.1107/S1600536810001352/im2174Isup2.hkl

Additional supplementary materials:  crystallographic information; 3D view; checkCIF report

## Figures and Tables

**Table 1:** Hydrogen-bond geometry (Å, °)

*D*—H⋯*A*	*D*—H	H⋯*A*	*D*⋯*A*	*D*—H⋯*A*
C3—H3*A*⋯O3^i^	0.95	2.50	3.423 (1)	165

Symmetry code: (i) 
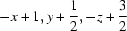
.

**Fig. 1.**
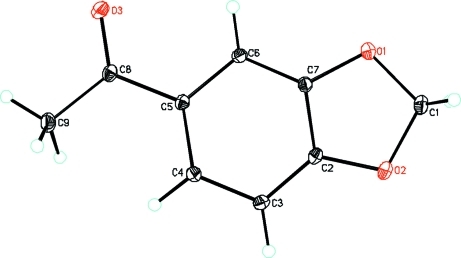
Molecular structure of (I), C9H8O3, showing the atom labeling scheme and 50% probability displacement ellipsoids.

**Fig. 2.**
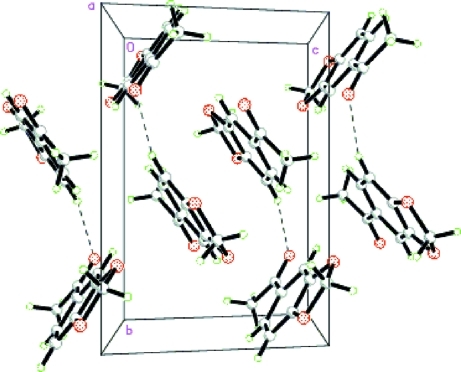
The molecular packing for (I) viewed down the a axis. Dashed lines indicate weak C—H···O intermolecular hydrogen bond interactions which link the molecule into chains propagating along the [011].

## supplementary crystallographic information

### Comment

Acetophenone is the simplest aromatic ketone. It is used as a polymerization catalyst for the manufacture of olefins, as an intermediate for pharmaceuticals, agrochemicals and other organic compounds, as a drug to induce sleep and as a solvent for plastics, resins, cellulose ethers, and esters. Acetophenone and its derivatives are ingredients of flavor and fragrance for soaps, detergents, cosmetics, and perfumes as well as in foods, beverages, and tobacco. Many synthetic or naturally occurring compounds containing the 1,3-dioxolyl group are very important because of their pharmacological properties (Ma *et al.* 1987*a*,*b*; Ohta & Kimoto 1976; Krause & Goeber 1972; Gabrielsen *et al.* 1992). The crystal structure of 1,3-benzodioxol-5-ylmethanol (Yathirajan *et al.*, 2007) is reported. The title compound, (I), was used recently for the synthesis of (2E)-1-(1,3-benzodioxol-5-yl)-3-(4-chlorophenyl)prop-2-en-1-one and (2E)-1-(1,3-benzodioxol-5-yl)-3-(3,4-dimethoxyphenyl) prop-2-en-1-one (Jasinski *et al.*, 2008). In view of the importance of the title compound, C_9_H_8_O_3_, (I), we report the crystal structure.

The molecular structure consists of an ethanoyl group bonded to a benzene group which is fused to a 1,3-dioxol ring in a nearly planar fashion (Fig. 1). The dihedral angle between the mean planes of the benzene and dioxol ring is 1.4 (8)°, as the dioxol group maintains itself in a slightly distorted envelope configuration (Cremer & Pople, 1975) with puckering parameters Q(2) and Phi(2) of 0.1020 and 34.7750, respectively. For an ideal envelope, Phi(2) has a value of k *x* 36. Bond lengths and bond angles are all within expected ranges (Allen *et al.* 1987).

Weak intermolecular C—H···O hydrogen bond interactions link the molecules into chains propagating in the [011] direction (Fig. 2). Crystal packing exhibits weak *Cg*2—*Cg*2 π-π interactions as evidenced by relatively short distances between the centroids of nearby aromatic rings (*Cg*2—*Cg*2: 3.8019 Å; slippage = 1.630 Å; 1 - *x*, -*y*, -*z*; *Cg*2 = ring centroid for C2—C7). A geometry optimized MOPAC AM1 computational calculation (Schmidt & Polik 2007) on (I) (AM1 (Austin Model 1 approximation), *in vacuo*, results in a completely planar molecule. This observation supports a suggestion that intermolecular forces influence the molecular conformation in the crystal.

### Experimental

The title compound (I) was obtained from Aldrich Chemical Company and was recrystallized from DMF by slow evaporation (m.p.: 360–362 K). Analysis for the title compound C_9_H_8_O_3_: Found (calculated): C: 65.85 (65.91); H: 4.91(4.86).

### Refinement

All H atoms were placed in calculated positions and wer refined using the riding model with C—H = 0.95–0.98 Å, and with *U*_iso_(H) = 1.17–1.50*U*_eq_(C).

### Crystal data

**Table:**

C_9_H_8_O_3_	*F*(000) = 344
*M**_r_* = 164.15	*D*_x_ = 1.470 Mg m^−^^3^
Monoclinic, *P*2_1_/*c*	Mo *K*α radiation, λ = 0.71073 Å
Hall symbol: -P 2ybc	Cell parameters from 5168 reflections
*a* = 9.4697 (3) Å	θ = 4.8–34.7°
*b* = 10.8445 (3) Å	µ = 0.11 mm^−^^1^
*c* = 7.5148 (3) Å	*T* = 200 K
β = 105.973 (3)°	Irregular plate, colorless
*V* = 741.93 (4) Å^3^	0.58 × 0.45 × 0.26 mm
*Z* = 4	

### Data collection

**Table:**

Oxford Diffraction R Gemini diffractometer	3061 independent reflections
Radiation source: fine-focus sealed tube	2215 reflections with *I* > 2σ(*I*)
graphite	*R*_int_ = 0.024
Detector resolution: 10.5081 pixels mm^-1^	θ_max_ = 34.8°, θ_min_ = 4.9°
φ and ω scans	*h* = −14→14
Absorption correction: multi-scan (*CrysAlis RED*; Oxford Diffraction, 2007)	*k* = −17→15
*T*_min_ = 0.909, *T*_max_ = 0.972	*l* = −11→11
12470 measured reflections	

### Refinement

**Table:**

Refinement on *F*^2^	Primary atom site location: structure-invariant direct methods
Least-squares matrix: full	Secondary atom site location: difference Fourier map
*R*[*F*^2^ > 2σ(*F*^2^)] = 0.045	Hydrogen site location: inferred from neighbouring sites
*wR*(*F*^2^) = 0.133	H-atom parameters constrained
*S* = 1.03	*w* = 1/[σ^2^(*F*_o_^2^) + (0.0852*P*)^2^] where *P* = (*F*_o_^2^ + 2*F*_c_^2^)/3
3061 reflections	(Δ/σ)_max_ < 0.001
110 parameters	Δρ_max_ = 0.39 e Å^−^^3^
0 restraints	Δρ_min_ = −0.28 e Å^−^^3^

### Special details

**Table:**

Geometry. All e.s.d.'s (except the e.s.d. in the dihedral angle between two l.s. planes) are estimated using the full covariance matrix. The cell e.s.d.'s are taken into account individually in the estimation of e.s.d.'s in distances, angles and torsion angles; correlations between e.s.d.'s in cell parameters are only used when they are defined by crystal symmetry. An approximate (isotropic) treatment of cell e.s.d.'s is used for estimating e.s.d.'s involving l.s. planes.
Refinement. Refinement of *F*^2^ against ALL reflections. The weighted *R*-factor *wR* and goodness of fit *S* are based on *F*^2^, conventional *R*-factors *R* are based on *F*, with *F* set to zero for negative *F*^2^. The threshold expression of *F*^2^ > σ(*F*^2^) is used only for calculating *R*-factors(gt) *etc*. and is not relevant to the choice of reflections for refinement. *R*-factors based on *F*^2^ are statistically about twice as large as those based on *F*, and *R*- factors based on ALL data will be even larger.

### Fractional atomic coordinates and isotropic or equivalent isotropic displacement parameters (Å^2^)

**Table:**

	*x*	*y*	*z*	*U*_iso_*/*U*_eq_	
O1	0.19844 (7)	0.26081 (7)	0.45409 (11)	0.03260 (19)	
O2	0.12787 (7)	0.42705 (7)	0.60401 (10)	0.02944 (17)	
O3	0.76739 (8)	0.26971 (7)	0.62415 (11)	0.03273 (18)	
C1	0.07627 (10)	0.31692 (10)	0.50175 (15)	0.0315 (2)	
H1A	−0.0023	0.3370	0.3882	0.038*	
H1B	0.0357	0.2596	0.5777	0.038*	
C2	0.27739 (9)	0.42276 (8)	0.64441 (11)	0.02067 (17)	
C3	0.37564 (9)	0.50450 (8)	0.74958 (12)	0.02310 (18)	
H3A	0.3448	0.5732	0.8079	0.028*	
C4	0.52406 (9)	0.48140 (8)	0.76650 (12)	0.02156 (17)	
H4A	0.5960	0.5353	0.8397	0.026*	
C5	0.56965 (8)	0.38161 (8)	0.67914 (11)	0.01868 (16)	
C6	0.46499 (9)	0.30025 (8)	0.56785 (12)	0.02042 (17)	
H6A	0.4936	0.2329	0.5048	0.024*	
C7	0.32064 (9)	0.32334 (8)	0.55556 (11)	0.02051 (17)	
C8	0.72805 (9)	0.35800 (8)	0.70045 (12)	0.02201 (18)	
C9	0.84055 (10)	0.44275 (10)	0.82066 (14)	0.0293 (2)	
H9A	0.9373	0.4250	0.8032	0.044*	
H9B	0.8436	0.4298	0.9507	0.044*	
H9C	0.8139	0.5286	0.7863	0.044*	

### Atomic displacement parameters (Å^2^)

**Table:**

	*U*^11^	*U*^22^	*U*^33^	*U*^12^	*U*^13^	*U*^23^
O1	0.0185 (3)	0.0348 (4)	0.0419 (4)	−0.0031 (3)	0.0041 (3)	−0.0136 (3)
O2	0.0162 (3)	0.0337 (4)	0.0373 (4)	0.0036 (2)	0.0055 (3)	−0.0046 (3)
O3	0.0236 (3)	0.0311 (4)	0.0461 (4)	0.0029 (3)	0.0140 (3)	−0.0052 (3)
C1	0.0180 (4)	0.0394 (5)	0.0362 (5)	−0.0029 (4)	0.0061 (4)	−0.0063 (4)
C2	0.0166 (3)	0.0237 (4)	0.0217 (4)	0.0032 (3)	0.0054 (3)	0.0019 (3)
C3	0.0227 (4)	0.0221 (4)	0.0248 (4)	0.0030 (3)	0.0071 (3)	−0.0028 (3)
C4	0.0204 (4)	0.0209 (4)	0.0227 (4)	−0.0010 (3)	0.0048 (3)	−0.0013 (3)
C5	0.0172 (3)	0.0191 (4)	0.0201 (4)	0.0006 (3)	0.0056 (3)	0.0024 (3)
C6	0.0203 (4)	0.0188 (4)	0.0230 (4)	0.0013 (3)	0.0075 (3)	−0.0009 (3)
C7	0.0178 (3)	0.0210 (4)	0.0217 (4)	−0.0014 (3)	0.0036 (3)	−0.0005 (3)
C8	0.0186 (3)	0.0227 (4)	0.0261 (4)	0.0004 (3)	0.0084 (3)	0.0039 (3)
C9	0.0186 (4)	0.0328 (5)	0.0353 (5)	−0.0031 (3)	0.0056 (3)	−0.0003 (4)

### Geometric parameters (Å, °)

**Table:**

O1—C7	1.3765 (10)	C4—C5	1.3945 (12)
O1—C1	1.4370 (12)	C4—H4A	0.9500
O2—C2	1.3648 (10)	C5—C6	1.4157 (11)
O2—C1	1.4314 (12)	C5—C8	1.4862 (11)
O3—C8	1.2256 (11)	C6—C7	1.3676 (11)
C1—H1A	0.9900	C6—H6A	0.9500
C1—H1B	0.9900	C8—C9	1.5053 (12)
C2—C3	1.3679 (12)	C9—H9A	0.9800
C2—C7	1.3879 (12)	C9—H9B	0.9800
C3—C4	1.3985 (11)	C9—H9C	0.9800
C3—H3A	0.9500		
			
C7—O1—C1	105.37 (7)	C4—C5—C8	121.13 (7)
C2—O2—C1	105.72 (7)	C6—C5—C8	118.56 (7)
O2—C1—O1	107.93 (7)	C7—C6—C5	116.75 (8)
O2—C1—H1A	110.1	C7—C6—H6A	121.6
O1—C1—H1A	110.1	C5—C6—H6A	121.6
O2—C1—H1B	110.1	C6—C7—O1	128.28 (8)
O1—C1—H1B	110.1	C6—C7—C2	122.11 (8)
H1A—C1—H1B	108.4	O1—C7—C2	109.56 (7)
O2—C2—C3	127.26 (8)	O3—C8—C5	120.79 (8)
O2—C2—C7	110.18 (7)	O3—C8—C9	120.11 (8)
C3—C2—C7	122.51 (8)	C5—C8—C9	119.09 (8)
C2—C3—C4	116.31 (8)	C8—C9—H9A	109.5
C2—C3—H3A	121.8	C8—C9—H9B	109.5
C4—C3—H3A	121.8	H9A—C9—H9B	109.5
C5—C4—C3	121.99 (8)	C8—C9—H9C	109.5
C5—C4—H4A	119.0	H9A—C9—H9C	109.5
C3—C4—H4A	119.0	H9B—C9—H9C	109.5
C4—C5—C6	120.31 (7)		
			
C2—O2—C1—O1	−10.84 (10)	C5—C6—C7—C2	1.19 (13)
C7—O1—C1—O2	10.94 (10)	C1—O1—C7—C6	175.70 (9)
C1—O2—C2—C3	−175.94 (9)	C1—O1—C7—C2	−6.94 (10)
C1—O2—C2—C7	6.63 (10)	O2—C2—C7—C6	177.78 (8)
O2—C2—C3—C4	−178.36 (8)	C3—C2—C7—C6	0.21 (14)
C7—C2—C3—C4	−1.22 (13)	O2—C2—C7—O1	0.22 (10)
C2—C3—C4—C5	0.83 (13)	C3—C2—C7—O1	−177.35 (8)
C3—C4—C5—C6	0.55 (13)	C4—C5—C8—O3	179.58 (8)
C3—C4—C5—C8	−179.39 (8)	C6—C5—C8—O3	−0.36 (12)
C4—C5—C6—C7	−1.54 (12)	C4—C5—C8—C9	0.81 (12)
C8—C5—C6—C7	178.40 (7)	C6—C5—C8—C9	−179.12 (8)
C5—C6—C7—O1	178.26 (8)		

### Hydrogen-bond geometry (Å, °)

**Table:**

*D*—H···*A*	*D*—H	H···*A*	*D*···*A*	*D*—H···*A*
C3—H3A···O3^i^	0.95	2.50	3.423 (1)	165

Symmetry codes: (i) −*x*+1, *y*+1/2, −*z*+3/2.
